# Evaluating Psychometric Properties of the Sultan Bin Abdulaziz Humanitarian City (SBAHC) Nurses’ Electronic Handover Tool

**DOI:** 10.7759/cureus.40026

**Published:** 2023-06-06

**Authors:** Ahmad S Al Baker, Fatima Ibrahim, Lina M Obaid, Febee Rose Obdamen, Ashraff Dimaocor

**Affiliations:** 1 Department of Nursing, Sultan Bin Abdulaziz Humanitarian City, Riyadh, SAU

**Keywords:** reliability, validity, perceptions, barriers, nursing, handover

## Abstract

Introduction: Handover is crucial during the transfer of patient care between healthcare professionals.Successful handover maintains patients' safety and high-quality care. The electronic handover of patients’ information is achievable, feasible, and potentially improves the quality of patient care. However, the introduction of the electronic handover is relatively recent and challenging for healthcare providers, especially nurses.

Objectives: Since Sultan Bin Abdulaziz Humanitarian City (SBAHC) has recently implemented an electronic handover system among nurses, the main aim of the current study was to develop a tool to assess the perception and barriers to electronic handover systems among nurses working at SBAHC and evaluate its psychometric properties.

Methods: The tool content and face validity were assessed by the content validity ratio (CVR). Exploratory and confirmatory factor analysis were used as validity methods, while the test-retest and inter-item consistency were used as reliability measures. The sample size was planned as five times the number of questions and a total of 200 nurses participated in the study.

Results: The criteria required for factor analysis were met as indicated by the results of the Kaiser-Mayer-Olkin test and Bartlett’s test of sphericity. The reliability results showed a Cronbach's alpha coefficient ranging from 0.858 to 0.910 for the perception subscale and from 0.564 to 0.789 for the barrier subscale, and an overall interclass correlation of 0.986, p<0.001.

Conclusions: The developed SBAHC electronic handover tool was valid and reliable, and it is advisable to consider it at the initial stages of implanting an electronic handover system to identify obstacles that are faced by the staff to be considered and addressed by the higher management.

## Introduction

Handover is crucial during the transfer of patient care between healthcare professionals [1,2]. Successful handover should maintain patients' safety and high quality of care. The handover process could be in verbal, written, or voice-recorded forms [3]. Several research studies have indicated that the handover process is usually associated with several problems, mainly high variability in the type of information that could lead to inefficiencies in clinical practice and suboptimal patient care [4]. Inappropriate handovers have many consequences, including, but not limited to, disruption of care, compromised patients' safety in the form of inappropriate drug dosing, surgeries on the wrong side, or even life-threatening side effects [5].

Handover is a significant nursing task, and it was estimated that nurses were involved in 40-70% of the total transfer and discharge handovers of patients in hospitals [6]. Therefore, it has been proposed that standardizing the content and processes of patient handovers would ensure consistency in the exchange of vital information. This would effectively improve communication and, consequently, patient safety [7]. Health information technologies such as electronic handover (e-handover) platforms are being increasingly utilized to overcome the challenges mentioned above in healthcare [8].

Several studies have shown that the electronic handover of patients is achievable, feasible, and potentially improves patient care [9,10]. It improved the transfer of care for many disciplines [11] and was associated with reduced rates of medication prescribing errors, hospital length of stay, and hospital mortality [12]. However, implementing an electronic handover system can be a major challenge, including technical factors such as numerous data entry fields, making it time-consuming and inefficient to use [13]. Additionally, the introduction of the electronic handover is relatively recent and represents a change in the routine of healthcare providers, especially nurses [14].

Recently, Sultan Bin Abdulaziz Humanitarian City (SBAHC) has implemented an electronic handover system to support an organized and high-quality handover process with an organized sequence of information with a timeliness patient-centered approach. However, as part of the quality improvement approach and the best utilization of allocated resources, the nursing department in SBAHC was interested in assessing the nurses' perceptions and barriers to the newly implemented electronic handover system. Therefore, the main aim of the current study was to develop a scale to assess the perception and barriers to electronic handover systems among nurses working in a tertiary rehabilitation center and evaluate its psychometric properties.

## Materials and methods

Study design

This was a cross-sectional study that was conducted from February to March 2022 and was conducted in two phases. The first phase was utilized to develop the questionnaire on the nurses' perceptions and barriers to using the electronic handover, followed by the second phase, where its validity and reliability were tested.

Phase 1: Development of the Tool

The research team designed a questionnaire to examine the perceptions and barriers of the nursing staff in using the new electronic handover practice. The questionnaire focused on effectiveness, usefulness, easiness, and barriers to using it. To increase the questionnaire's face validity, a group of experts including clinicians discussed and reviewed the appropriateness of the questionnaire items. Two experienced nurses made the first version of the questionnaire, and two other group members with experience in clinical and research fields were requested to report their comments. This process was repeated three times till the agreement of all the expert group members with all the items of the questionnaire (Appendices, Table 8, Supplementary 1).

Scoring of the tool: The response categories were identified on a 5-point Likert scale (1 = strongly agree, 2 = agree, 3 = neutral, 4 = disagree, 5 = strongly disagree), as shown in Supplementary 1 (Appendices, Table 8), SBAHC Nurses’ Electronic Handover Tool.

Piloting the tool: The tool was piloted among 96 nurses to assess the practicality of the tool and to estimate the time needed to complete it.

Phase 2: Evaluation of the Tool

In this phase, the face and content validity, the construct validity, and the reliability of the developed scale were checked. Face and content validity were assessed using the content validity index (CVI) and content validity ratio (CVR). Construct validity was assessed using factor analysis, while Cronbach's alpha tested internal consistency. In addition, the time variance of the tool was tested using the test-retest approach.

Sample and setting

An online survey was conducted from February to March 2022 to assess the validity of the developed scale. The participants were registered nurses employed in SBAHC. For explanatory factor analysis (EFA), previous studies have suggested that the number of required data items should be at least five times per item [15], and for building a structural equation model for confirmatory factor analysis (CFA), at least 200 samples are considered adequate [16]. According to these criteria, data from 200 nurses were collected.

Data collection procedures

All the nursing staff received the questionnaire in a Google form, a secure web-based form. The questionnaire consisted of 19 questions, which covered different topics, including questions on perceptions of using the electronic handover in terms of perceived effectiveness, usefulness, and easiness, along with barriers to using it.

Data analysis

The collected data were analyzed using Statistical Product and Service Solutions (SPSS) (IBM SPSS Statistics for Windows, Version 26.0, Armonk, NY), and the structural equation model (SEM) results were obtained using IBM SPSS AMOS 22.0 (IBM, NY, USA). Descriptive statistics were conducted, where categorical variables were presented as counts and proportions, while continuous variables were presented as mean and standard deviation (SD). The Chi-square test (χ2) was used for categorical data, while the unpaired t-test was used for numerical data.

In the face and content validity, the two indicators of CVR and CVI were quantitatively calculated. To calculate the CVR, the experts rated their opinions about the necessity of each item on a 3-point Likert format (necessary, helpful but not necessary, and not necessary). The obtained ratios for each item of the questionnaire were compared with the numbers given in the Lawashe table [17], and values > 0.62 were considered acceptable. To ensure the scale's relevance, clarity, and simplicity, the CVI was used using a three-point Likert format for each item based on the Lynn pattern [18]. The content validity of the questionnaire was confirmed if the score of CVI was higher than 0.79, while if it was between 0.70 and 0.79, it meant that it needed a revision, and if the CVI was less than 0.70, the item should be excluded [19].

For EFA, the maximum likelihood method was used to extract meaningful structures. Factor rotation was performed using the varimax rotation to facilitate the interpretation of the factor structure. The Kaiser-Meyer-Olkin (KMO) measure and Bartlett's test for sphericity were conducted to determine whether the collected data were suitable for factor analysis. In CFA, maximum likelihood estimation was used to estimate the CFA, and the criterion for a standardization factor load of 0.50 or greater was applied [20]. The χ2 statistics (p-value<o.o5), Chi-square minimum/degree of freedom (CMIN/DF) < 3.0, comparative fit index (CFI), the goodness of fit index (GFI), adjusted goodness-of-fit index (AGFI), and root mean square error of approximation (RMSEA) were used to calculate the fit indices. The reference values for each fitness index were p > .05 for chi-square, RMSEA ≤ 0.10, CFI > 0.90, GFI > 0.90, and AGFI > 0.80. The internal reliability of the developed tool was assessed by Cronbach's α, the internal consistency coefficient, while the external reliability was assessed through the test-retest approach.

## Results

Sample characteristics

The participants were mostly females at a rate of 65% with a mean age of 34.81±6.44 years and a mean duration for work experience of 10.07± 4.89 years. In addition, 50% of the nurses worked in the inpatient adult department, with the vast majority having a bachelor's degree (92%) and good computer skills (74.5%).

Validity and reliability of the developed scale

Face and Content Validity

After item generation, a total of 30 questions were designed, This was followed by qualitative deletion and correction through the face and content validity for the questions ending up with 19 items-questionnaire. The CVI value of the questionnaire was 0.95, with CVR ranging from 0.79 to 0.9, as shown in Table 1.

**Table 1:** The content validity ratio (CVR) values of each question

Construct	Statement	CVR
Perception		
Easy Use	Electronic handover is easy	0.92
	Electronic handover is practical	0.94
	Electronic handover is understandable	0.91
	Electronic handover is presented in an organized manner	0.90
Effectiveness	Electronic handover is effective in the transfer of information between nurses	0.91
	The electronic handover provides specific patients information	0.92
	The current electronic handover information system allows modification of the information	0.96
Reliability	Satisfied with the use of electronic handover	0.93
	The use of electronic handover is in accordance with the hospital’s legal policy.	0.89
	The information presented in the electronic handover is reliable	0.94
Barriers		
Practicality	Experience technical difficulties in using electronic handover information system	0.93
	Challenge in understanding the language used in electronic handover	0.83
	Difficulties in navigating the electronic handover	0.87
	Difficulty in retrieving the data from the electronic handover.	0.91
Knowledge/training	The training received on the proper utilization of electronic handover is not sufficient	0.95
	Awareness about the new hospital policy and procedures related to electronic handover	0.93
Technical knowledge	Technical downtime greatly affects the use of electronic handover.	0.91
	An electronic handover information system is not practical to use.	0.92
	The different delivery method affects the overall process of the electronic handover.	0.79

Construct validity

Exploratory Factor Analysis (EFA)

EFA was conducted for the 19 items. In this study, the KMO measure was 0.931, and Bartlett's sphericity was statistically significant (χ2 = 951.226, df = 45, p < .0001), which indicates that samples met the criteria for factor analysis. The perceptions part of the scale was composed of three factors: easiness of use, efficacy, and reliability. The percentage of factors explaining the total variance for the perception subscale was 42.11%, 51.94%, and 34.73% for factors 1, 2, and 3, respectively. Additionally, the part of the barrier was composed of three factors: technical challenges, practicality, and training and knowledge. The percentage of factors explaining the total variance for the barrier's subscale was 39.31%, 35.97%, and 32.20% for factors 1, 2, and 3, respectively. The limit value was 0.50 or greater for the load values for each factor. The results of EFA for the electronic handover perception and barriers scale are presented in Table 2.

**Table 2:** The goodness of fit indecencies for nurses’ perception subscale χ2: Chi-square; RMSEA: root mean square error of approximation; df: degree of freedom; GFI: goodness of fit index; AGFI: adjusted goodness-of-fit index; CFI: comparative fit index

Scale	χ2	df	RMSEA	GFI	AGFI	CFI
Perception of the electronic handover	136.181	32	0.09	0.91	0.85	0.95

Confirmatory Factor Analysis (CFA)

**Figure 1:**
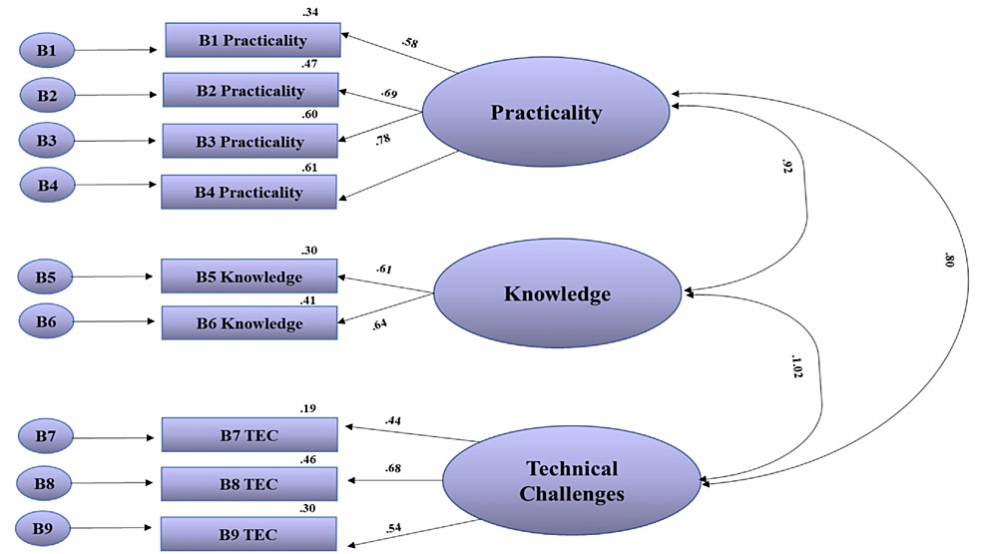
Confirmatory factor analysis results of the perception subscale of SBAHC nurses’ electronic handover tool SBAHC: Sultan Bin Abdulaziz Humanitarian City

As shown in Figure 1, the perception subscale consisted of 10 items and three factors (Table 1). The fit indices were as follows the Chi-square goodness of fit (χ2) = 91.181, df = 32, χ2/df = 2.8, RMSEA = 0.09, CFI = 0.95, GFI = 0.91, AGFI = 0.85, and IFI = 0.95 which indicated that the scale is at a sufficient level of fit. The regression coefficients and t values are given in Table 5, where the obtained values were significant, and the model is confirmed (Figure 1 and Table 3).

**Table 3:** The goodness of fit indecencies for nurses’ perception subscale χ2: Chi-square; RMSEA: root mean square error of approximation; df: degree of freedom; GFI: goodness of fit index; AGFI: adjusted goodness-of-fit index; CFI: comparative fit index

Scale	χ2	df	RMSEA	GFI	AGFI	CFI
Perception of the electronic handover	136.181	32	0.09	0.91	0.85	0.95

**Figure 2:**
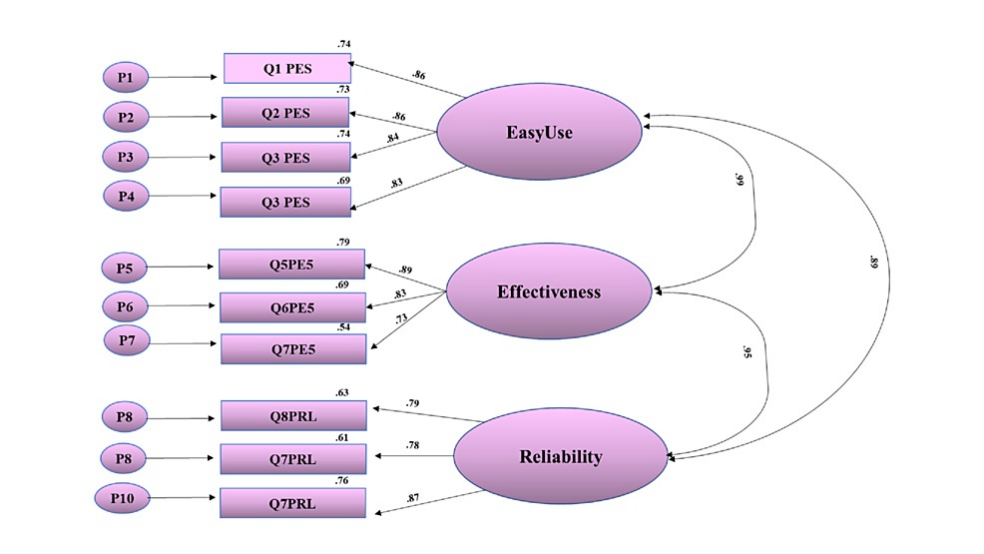
Confirmatory factor analysis results of the barriers subscale of SBAHC nurses’ electronic handover tool SBAHC: Sultan Bin Abdulaziz Humanitarian City

The barriers subscale consisted of nine items and three factors (Table 1). The fit indices were as follows the Chi-square goodness of fit (χ2) = 91.181, df = 32, χ2/df = 2.8, RMSEA = 0.09, CFI = 0.95, GFI = 0.91, AGFI = 0.85, and IFI = 0.95 which indicated that the scale is at a sufficient level of fit (Figure 2 and Table 4).

**Table 4:** The goodness of fit indecencies for nurses’ barrier subscale χ2: Chi-square; RMSEA: root mean square error of approximation; df: degree of freedom; GFI: goodness of fit index; AGFI: adjusted goodness-of-fit index; CFI: comparative fit index

Scale	χ2	df	RMSEA	GFI	AGFI	CFI
Perception of the electronic handover	73.079	24	0.09	0.91	0.85	0.95

The regression coefficients and t values are given in Table 5 and Table 6 where the obtained values were significant, and the model is confirmed.

**Table 5:** The regression and t values of the confirmatory factor analysis for the perception subscale

Items	Regression values	t values
Item 1	0.858	15.928
Item 2	0.856	15.914
Item 3	0.842	15.448
Item 4	0.830	15.075
Item 5	0.828	15.345
Item 6	0.888	16.056
Item 7	0.735	12.965
Item 8	0.792	11.831
Item 9	0.782	11.944
Item 10	0.870	13.667

**Table 6:** The regression and t values of the confirmatory factor analysis for the barrier’s subscale

Items	Regression values	t values
Item 1	0.583	9.42
Item 2	0.687	11.35
Item 3	0.775	12.83
Item 4	0.780	12.94
Item 5	0.640	10.40
Item 6	0.437	8.68
Item 7	0.681	11.73
Item 8	0.615	11.64
Item 9	0.545	7.402

Reliability

Internal Consistency

As shown in Table 7, Cronbach's alpha coefficient of the two subscale constructs was calculated, and all the constructs had good reliability ranging from 0.858 to 0.910 for the perception subscale and from 0.564 to 0.789 for the barrier subscale.

**Table 7:** Internal consistency of the nurses’ perceptions and barriers of electronic handover scale

Construct	Statement	Cronbach’s Alpha
Perception		
Easy Use		0.910
Q1	Electronic handover is easy	
Q2	Electronic handover is practical	
Q3	Electronic handover is understandable	
Q6	Electronic handover is presented in an organized manner	
Effectiveness		0.8771
Q4	Electronic handover is effective in the transfer of information between nurses	
Q8	The electronic handover provides specific patients information	
Q9	The current electronic handover information system allows modification of the information	
Reliability		0.858
Q5	Satisfied with the use of electronic handover	
Q7	The use of electronic handover is in accordance with the hospital’s legal policy.	
Q10	The information presented in the electronic handover is reliable	
Barriers		
Practicality		0.789
Q11	Experience technical difficulties in using electronic handover information system	
Q12	Challenge in understanding the language used in electronic handover	
Q13	Difficulties in navigating the electronic handover	
Q14	Difficulty in retrieving the data from the electronic handover.	
Knowledge/training		0.564
Q15	The training received on the proper utilization of electronic handover is not sufficient	
Q18	Awareness about the new hospital policy and procedures related to electronic handover	
Technical knowledge		0.614
Q16	Technical downtime greatly affects the use of electronic handover.	
Q17	An electronic handover information system is not practical to use.	
Q19	The different delivery method affects the overall process of the electronic handover.	

Time-invariance analysis

A paired t-test was carried out approximately 30 days later. There was no significant difference between the pre-test and post-test results (p = 0.74). However, when Pearson's correlation analysis examined the scores obtained from the first and second measurements, there was a strong positive correlation between the scores of the two measurements with the 30 days interval (r = 0.986, p<0.001).

## Discussion

In a healthcare setting, electronic handover platforms are being increasingly utilized to overcome the logistical challenges of healthcare. However, the changeover from traditional handover methods to electronic handover is tremendously stressful for nurses and other healthcare providers. Assessing nurses' perceptions and barriers to the implementation of an electronic handover system is crucial to identify obstacles that might negatively affect the handover process and consequently affect the continuity of care. Additionally, the results of such assessment could serve as a reference for measures and interventions that could be implemented to facilitate the nurses’ adaptation to the electronic handover system.

The current study assessed the psychometric properties of the developed nurses’ perceptions and barriers of the electronic handover scale that consisted of 19 items including items for the perception subscale and nine items for the barrier subscale. Each one has three factors, whose fit indices indicated that the scale is at an acceptable level of fit. Factor analysis is the most commonly used method for testing scale validity. Even though there are several goodness of fit indices for CFA, the chi-square/df, RMSEA, AGFI, GFI, and CFI results are reported as the most commonly reported fit indices in validity analysis studies [21]. Additionally, it is recommended to evaluate KMO and Bartlett’s test to assess the adequacy of the sample and appropriateness of the factor correlation matrix before conducting factor analysis [22]. In this study, the results from CFA and the EFA for the developed scale have shown that the scale is sufficiently strong to assess the nurses’ perception and barriers to electronic handover.

The reliability coefficients and internal consistency values as well as the test-retest values showed that the SBAHC electronic handover tool was reliable. Reliabilities for the three subscales namely, easy use, effectiveness, and reliability were excellent, indicating that quality of information and interaction and support were reliable and valid measures of perceptions in using the electronic handover. Although the mean inter-item correlation for the three-item subscale was within acceptable limits, additional research into the impact of including additional items on the perception of reliability for the three-item subscale would be beneficial.

The SBAHC electronic handover tool is a valid and reliable self-reported measure of the perception and barriers of the electronic handover process. This scale will help highlight the areas that need intervention to improve the adaptation and acceptance of the nurses to the new handover technique. Since the quality of handover is an important aspect of care, it must be monitored and evaluated, and development as a tool for monitoring and evaluating handover processes is merit. The electronic handover can help organizations ensure safe patient care by identifying areas that need education and development in the handover process.

## Conclusions

Handover is a crucial aspect of patient care, and the quality of the handover process is a major component of the quality of the patient’s care. Electronic handover is feasible and has the potential to improve patient care. The developed SBAHC electronic handover tool was valid and reliable, and it is advisable to consider it at the initial stages of implanting an electronic handover system to identify obstacles that are faced by the staff to be considered and addressed by the higher management. Further studies should assess the impact of the electronic handover on the quality of patients' care and patient satisfaction.

## Appendices

**Table 8:** Supplementary 1: SBAHC nurses’ electronic handover tool The perception subscale consisted of 10 items (Q1-Q10) and three factors (easy use, effectiveness, and reliability). The barriers subscale consisted of nine items (Q11-Q19) and three factors (practicality, knowledge/training, and technical knowledge). The response categories were identified on a 5-point level of agreement (1 = strongly agree, 2 = agree, 3 = neutral, 4 = disagree, 5 = strongly disagree). SBAHC: Sultan Bin Abdul-Aziz Humanitarian City

Item		Strongly agree	Agree	Neutral	Disagree	Strongly disagree
Q1	Electronic handover is easy.					
Q2	Electronic handover is practical.					
Q3	Electronic handover is understandable.					
Q4	Electronic handover is effective in the transfer of information between nurses.					
Q5	Satisfied with the use of electronic handover.					
Q6	Electronic handover is presented in an organized manner.					
Q7	Satisfied with the use of electronic handover.					
Q8	The electronic handover provides specific patient information.					
Q9	The current electronic handover information system allows modification of the information.					
Q10	The information presented in the electronic handover is reliable.					
Q11	Experience technical difficulties in using electronic handover information system					
Q12	Challenge in understanding the language used in electronic handover					
Q13	Difficulties in navigating the electronic handover					
Q14	Difficulty in retrieving the data from the electronic handover.					
Q15	The training received on the proper utilization of electronic handover is not sufficient.					
Q16	Technical downtime greatly affects the use of electronic handover.					
Q17	An electronic handover information system is not practical to use.					
Q18	Awareness about the new hospital policy and procedures related to electronic handover					
Q19	The different delivery method affects the overall process of the electronic handover.					
